# Supplementary material for: Are carbon emissions trading and green financial instruments synergistic? -Comprehensive quantitative research based on content analysis
Source: PLoS One. 2024 Mar 7;19(3):e0298601. doi: 10.1371/journal.pone.0298601 (PMC10919601; doi:10.1371/journal.pone.0298601)
Supplement: S1 Data — (ZIP) [file pone.0298601.s001.zip › Data/地级市Z_F工作报告.zip!/║■▒▒/╗╞╩»╩╨/└┤╫╘╣1⁄2╓┌║┼í╛┬φ┐╦╩2╛▌═°í┐_2016.docx]

各位代表：

现在，我代表市人民政府，向大会作工作报告，请连同《黄石市国民经济和社会发展第十三个五年规划纲要(草案)》一并审议，并请市政协委员和其他列席人员提出意见。

一、“十二五”时期回顾和2015年工作

“十二五”时期是黄石发展很不平凡的五年。面对错综复杂的宏观环境和艰巨繁重的改革发展任务，在省委、省政府和市委的正确领导下，全市上下深入实施“生态立市产业强市、加快建设现代化特大城市”战略，励精图治、奋力拼搏，“十二五”目标任务如期完成，城市竞争力和影响力跃上了新台阶。

——这五年，城市转型扎实推进，综合实力稳步提升。传统支柱产业改造升级，新兴产业蓬勃发展，现代服务业快速壮大，高新技术产业增加值占生产总值的比重比“十一五”末提高0.9个百分点。全市生产总值、地方公共财政预算收入分别跨越千亿元、百亿元大关，年均增长10.3%和19.8%；全社会固定资产投资累计完成4741亿元，年均增长23.8%。黄石连续11年被评为省级金融信用市。黄石开发区获批国家新型工业化示范基地，大冶市在全国县域经济百强县(市)排名88位，实现四年四进位。

——这五年，新型城镇化进程加快，城市发展从“环磁湖”向“环大冶湖”迈进。450平方公里的大冶湖生态新区建设如火如荼，中心城区与大冶、阳新加快融合，大路网、大港口、大物流体系加速构建，全市城镇化率达60.9%。

——这五年，改革开放不断深化，发展动力更加强劲。住房保障、工矿废弃地复垦利用、农村土地确权等重点领域改革取得新突破。全省地级市首家保税物流中心封关运行。实际利用外资、外贸出口年均分别增长15%和22.6%，黄石被评为“中国外贸百强城市”。

——这五年，美丽黄石建设不断加强，生态环境明显改善。“五城同创”扎实推进，节能减排取得重大进展，生态欠账加快偿还，“十二五”各项约束性指标全面完成。荣获全国国土资源节约集约模范城市、国家节水型城市，蝉联国家园林城市。成功举办两届市级园博会，绿色理念深入人心，“绿满黄石”持续升温，城市宜居度、美誉度不断提升。

——这五年，人民生活持续改善，社会事业快速发展。科技、教育、卫生、文化事业繁荣发展，社会公共服务体系基本建立，减少贫困人口8万多人，人民生活水平不断提高，黄石荣获全国创业先进城市、国家公共文化服务示范区、全国社区治理和服务创新实验区、全国救急难试点城市等称号。

——这五年，精神文明和民主法治建设不断加强，社会更加和谐稳定。深入推进精神文明创建活动，市民素质和城市文明程度进一步提升。扎实推进法治政府建设，主动接受市人大及其常委会的法律监督和工作监督，自觉接受市政协的民主监督和社会舆论监督，充分听取各民主党派、工商联的意见建议，认真办复人大代表建议和政协提案。加强与工会、共青团、妇联等群众团体联系。

与此同时，加强和创新社会治理，平安黄石建设深入推进，“六五”普法全面完成，安全生产、疾病防控和食品药品安全保障体系不断健全，国防教育和“双拥”工作不断加强，对台、信访、民族、宗教、保密、档案、妇女儿童、老龄、残疾人、援藏、援疆、援利川、统计、审计、外事、侨务、国家安全、科普、防震减灾、民防、气象、史志、法律援助等各项工作都取得新的成就。

2015年是“十二五”规划的收官之年。面对复杂严峻的宏观经济形势，我们坚持稳中求进的工作总基调和“竞进提质、升级增效”的工作总要求，坚持“打基础、管长远”不放松，坚持改革创新不动摇，经济社会发展实现深度转型、逆势而进。预计全市完成生产总值1220亿元，增长5.3%；全社会固定资产投资1380亿元，增长18.1%；社会消费品零售总额582.36亿元，增长12.1%；地方公共财政预算收入100.58亿元，增长12.5%；外贸出口16.64亿美元，增长15%；城镇和农村常住居民人均可支配收入分别增长9.2%和10.1%。一年来，主要抓了五个方面的工作：

(一)着力推进产业转型

加快振兴黄石制造。制定出台振兴“黄石制造”行动计划，明确了产业发展目标任务，设立产业发展基金，整合财政资金1亿元，引导企业实施技改和产业转型项目400多个，发放企业解困帮扶资金、新上技改项目补贴资金4395万元。积极对接国家重大产业振兴和技术改造项目专项，东贝、劲牌、新冶钢、大冶有色、华新水泥5家企业获得国家投资补助资金9784万元。大冶有色成功列入全国“城市矿产”基地。建立“一个产业、一个规划、一套政策、一支基金、一个园区、一个协会”工作推进机制，加快电子信息、装备制造、生物医药、智能模具等产业发展，汽车零部件产业成为省级重点成长型产业集群，电子信息产业产值保持50%以上的快速增长，黄石入选2015年宽带中国示范城市。大力推进产业链招商，新批外商投资企业9家，新注册重点项目84个，其中5亿元以上重大项目14个。加强项目建设，新冶钢中棒材等102个亿元以上项目竣工投产，欣兴电子、宏广电子等97个亿元以上项目开工建设。大力开展“千名干部进千企”活动，全力帮助企业度冬迎春。加强中小企业服务平台建设，市中小企业担保公司注册资本超过10亿元，共为近2000家中小微企业提供融资担保。实施中小企业成长工程，新进规企业102家。

大力发展现代服务业。设立现代服务业发展专项资金，通过市级投融资平台建立若干支产业基金，推动现代服务业快速发展。沃尔玛、万达广场、华中矿产品交易中心建成营业。一批重点项目、重点企业、知名品牌纳入全省服务业“五个一百”工程。积极打造工业旅游项目，精心培育旅游节庆品牌，乡村旅游精彩纷呈。全市实现服务业增加值420亿元，增长6.5%，第三产业增加值占GDP比重比上年提高2.4个百分点。

加快发展现代农业。新增菜地面积1万亩，初步形成万亩连片基地3个。新型农业经营主体加快发展，新增省级龙头企业5家、合作社221个、家庭农场115个，升级改造村级综合服务社100家。农产品电子商务蓬勃兴起。

(二)着力加强新区建设和旧城改造

大冶湖生态新区建设全面提速。把大冶湖生态新区作为黄石大城建设的主战场，加快推进核心区建设，“两纵一横”道路全面实施，黄阳快速路开工建设,月亮山隧道顺利贯通，园博园、矿博园、鄂东医疗中心等重点项目加快推进。引导社会资本进入城市基础设施建设领域，大冶湖生态新区核心区地下综合管廊等项目建设PPP模式全面启动。

着力完善城市功能。启动黄石2049城市远景发展战略研究。完成黄石基本生态控制线专项规划，规划展示馆建成开馆。着力抓好水、电、气、路、管网建设，改造中心城区易渍水点11处，疏通排水管网65公里，新增天然气管网35公里，新增天然气用户1.5万户，维护改造城市道路2.3万平方米。海绵城市、韧性城市建设深入实施。完成城市重大项目投资100亿元。

推进交通基础设施建设。棋盘洲长江公路大桥连接线、武九客专黄石段全线开工，兴国至富池一级公路、316国道分线改造等重点工程加快推进，棋盘洲长江公路大桥、武穴长江公路大桥通过国家发改委核准批复。站场建设力度加大，团城山客运枢纽站开工建设。进一步发展农村交通，新改建农村公路81.3公里，加宽路基1477公里，投入车辆256台，实现村村通客车。

加快城镇化和新农村建设。完成两镇一区“四化同步”规划，启动编制部分乡镇总体规划和农村居民点布局规划。打造了一批省级宜居村庄和新农村建设示范村。农村生活垃圾综合治理顺利实施。农村危房改造、渔民上岸与库区移民避险工程全面启动。投资7.19亿元，开工各类水利工程3100余处，更新改造和维修泵站53座，除险加固小Ⅱ型水库67座，解决16.82万农村居民和7.22万农村学校师生饮水安全问题。建成高标准基本农田9万亩，完成低丘岗地改造2.1万亩，完成县级占补平衡续建项目25个，新增耕地近万亩。扎实开展精准扶贫、精准脱贫，安排专项资金支持阳新扶贫攻坚。

提升城市管理水平。实施城市管理疏导工程，深入开展以违法建筑管控、市容综合治理、户外广告整治、渣土抛洒管控等为重点的专项整治活动。建立健全精细化管理、定期检查督办、媒体公开曝光等工作机制，有效提升城市管理效能。统筹推进“五城同创”，通过省级卫生城市复查和国家卫生城市省级考核，黄石再次、大冶首次荣获“全国文明城市”提名城市。大冶市通过国家园林城市考核。

(三)着力改善生态环境

加强生态环境治理。以山、地、路、车、店、厂为重点，积极开展大气污染专项整治，中心城区环境空气质量优良率超过70%。亚行贷款黄石水污染综合治理项目稳步推进，青山湖水污染治理进展顺利，磁湖湿地公园项目启动。土壤重金属污染综合治理工程加快实施。

加大生态建设力度。扎实开展“绿满黄石”行动，大力实施“五边三化”、“八园六带”工程，完成人工造林15.8万亩，新增改造城市绿地80公顷，建成城市生态绿道22公里，柯尔山-白马山生态公园主体工程完工，建成国家级绿色矿山8家，建成国家级生态乡镇2个、省级生态乡镇(村)30个，建成一批绿色学校、绿色社区，森林火灾得到有效遏制，被评为省级森林城市。成功纳入国家生态文明先行示范区建设行列。

着力推进节能减排。大力改善能源结构，发展循环经济，劲牌公司沼气发电等一批新能源项目开工建设。积极推进建筑节能改造，新建绿色建筑32万平方米，实现可再生能源建筑应用70万平方米。引导企业大力开展清洁生产，完成烟气脱硫、脱硝等减排项目69个。

(四)着力推进改革创新

全面深化改革。深入推进行政审批制度改革，制定并公布市、区行政审批通用目录。积极开展工商“同城通办”试点，实行商事登记“三证合一”，企业注册时间缩减到3个工作日以内。新一轮政府机构改革有序推进。深化投融资体制改革，成立市投资和债务管理委员会，重组市城投、交投、众邦、国资等投融资平台。组建市矿业投资公司，建立矿业权一级市场。推进政府购买服务、预算绩效和投资评审管理改革，完成“营改增”改革试点。多层次资本市场建设扎实推进，15亿元的城投债获批发行，2家企业登陆“新三板”，加强与武汉股权交易中心的战略合作，22家企业在湖北四板(科技板)挂牌，形成企业上市“黄石板块”。成功获批全国内贸流通体制改革发展综合试点城市。铁山区入选“国家独立工矿区转型试点”。成立鄂东医疗集团、鄂东职教集团，积极推进医药卫生、职业教育改革。国企、农村等领域改革有序推进，农村土地确权颁证基本完成。

大力推进“双创”工作。围绕打造创新之城、创业之都、创客之家，出台“双创”实施意见，“磁湖汇”众创空间正式运营。大力实施招才引智工程，引进各类急需紧缺人才3735人，分配人才公寓200套。实施科技企业创业与培育工程，设立科技创业与培育专项资金300万元、孵化器种子基金3000万元，各类科技企业孵化器达到16家，其中省级创新型孵化器4家，在孵企业近400家。帮扶创业7700人，带动就业2.68万人。新登记各类市场主体5.1万户，增长36.5%。着力增强科技创新能力，新增院士工作站1家、省级企业技术中心2家、省工程实验室3家。组织实施各类科技项目85项，向上争取科技项目41项。共登记省级重大科技成果113项，获得省级科技奖励12项。全市发明专利申请量、专利授权量均突破1000件。新增高新技术企业28家，总量突破100家。黄石大冶湖高新技术产业园区申报国家级高新区加快推进。全国质量强市示范城市创建工作扎实开展，新增中国驰名商标2件、地理标志1件。

进一步加快开放进程。深化与盐田港集团战略合作，黄石新港正式开港，标志着多式联运进入快车道。积极参与“一带一路”战略，华新水泥、十五冶等企业走出去取得新成效。棋盘洲保税物流中心辐射范围不断扩大，黄石商品保税展示交易中心建成营业。

(五)着力建设和谐社会

扎实抓好就业和社会保障。全市城镇新增就业7万人。积极服务企业用工，累计为企业招工2.76万人(次)。社会保障水平不断提高，五项社会保险参保总数达到248.46万人(次)，征缴社会保险费36.7亿元。顺利完成全市21.6万名企业退休人员养老金上调任务。城乡居民医保和新农合保障水平稳步提高，各级财政人均补助标准提高到380元，全市参合率达到99.8%。城乡低保两次提标，保障标准位居全省前列。城市居家养老服务实现全覆盖。全面减免城区居民基本殡葬费用。

大力推进棚户区改造。坚持把棚户区改造作为“一号民生工程”，科学制定三年改造计划，实行“政府指导、群众主导”，引导群众成立自改委，大力推进货币化安置，通过完善配套政策、搭建代筹代购平台等多项举措，统筹解决资金筹措、房屋征收、稳定房地产市场、化解社会矛盾等难点问题，初步形成棚改征收、腾退土地、金融造血、滚动发展格局。全年开工建设各类保障性住房、棚户区改造住房20943套，基本建成各类保障房15426套，分配入住19149套，新增租赁补贴350户。

统筹发展社会事业。推进义务教育均衡发展，筹集资金2.88亿元，积极改善办学条件，接收流动人口子女入学1.24万人。总投资11亿元的阳新教育城项目主体工程完工。基本公共卫生服务更加公平可及，公立医院改革扎实推进，基本药物制度实施范围不断扩展，人口和计划生育管理不断加强。文化惠民力度加大，全市“四馆一站”免费开放，广播电视村村通(户户通)、“村村响”、无线覆盖工程建设任务全面完成，建成农村文化礼堂297个，精品话剧《刘伦堂》在第二届湖北艺术节开幕式上首演。成功举办2015环中国国际公路自行车赛、全国半程马拉松邀请赛，新国乒基地正式运营，父子山、熊家境登山步道被命名为国家登山健身步道。

奋斗历经艰辛，成绩来之不易。这是市委正确领导的结果，是市人大、市政协监督支持的结果，是全市人民共克时艰、真抓实干的结果。在此，我代表市人民政府，向全市人民，向给予政府工作大力支持、有效监督的人大代表、政协委员，向民主党派、工商联、无党派人士，各人民团体，向驻黄解放军、武警官兵，向所有关心支持黄石建设发展的海内外人士，向所有在黄的投资者、创业者和建设者，表示衷心的感谢!

各位代表，五年的工作实践，我们深刻体会到：必须坚持战略引领，坚定不移实施“生态立市产业强市、加快建设现代化特大城市”发展战略；必须坚持项目支撑，狠抓招商引资项目建设，不断夯实转型发展底盘；必须坚持改革创新，扩大开放，为经济社会发展注入持久动力；必须坚持绿色发展，切实推进生态文明建设；必须坚持改善民生，不断提高人民群众的幸福感和获得感。

我们也十分清醒地认识到，发展中的深层次矛盾和问题仍然十分突出：发展不够、总量不大、结构不优仍是黄石最大的实际，城市竞争力与区域中心城市的要求还有一定差距；经济下行压力加大，实体经济发展困难；资源环境约束加剧，生态修复治理的压力依然很大；统筹发展不够，市域一体发展和改善民生工作有待继续加强；政府行政效能仍需提升，发展环境有待进一步优化。对此，我们将认真对待，采取有力措施，积极推进解决。二、“十三五”目标任务

“十三五”时期是我国全面建成小康社会的决胜阶段，也是黄石加快建设现代化特大城市的关键时期。必须高举中国特色社会主义伟大旗帜，全面贯彻党的十八大和十八届三中、四中、五中全会精神，以马克思列宁主义、毛泽东思想、邓小平理论、“三个代表”重要思想、科学发展观为指导，深入贯彻习近平总书记系列重要讲话精神，以“四个全面”战略布局为统领，牢固树立创新、协调、绿色、开放、共享的发展理念，坚持生态立市产业强市、加快建设现代化特大城市，推进经济社会深度转型，全面提升区域中心功能，统筹推进经济建设、政治建设、文化建设、社会建设、生态文明建设，在全省率先全面建成小康社会，为2049年全面建成现代化特大城市打下坚实基础。

“十三五”时期经济社会发展目标是：到2020年，实现地区生产总值、规模以上工业增加值、地方公共财政预算收入在2015年的基础上翻一番，固定资产投资五年累计过万亿元。努力把黄石建设成为鄂东区域性中心城市、长江经济带重要战略节点城市和国家生态文明先行示范区。

“十三五”的主要任务是：

(一)坚持创新发展，增强产业强市新动力。深入实施创新驱动发展战略，大力振兴“黄石制造”，加快建设全国重要电子信息产业基地、特钢和铜产品精深加工基地、生命健康产业基地、节能环保产业基地和中部地区先进制造业中心。推动服务业快速发展，建设区域性金融中心，打造长江中游大宗工业品交易中心、鄂东商贸中心和武汉城市圈重要旅游目的地城市。构建产业主导型创新体系，强化企业创新主导作用，大力推进“大众创业、万众创新”，充分激发全社会创新创业热情，加快建设创新型城市。

(二)坚持协调发展，构建市域发展新格局。强化区域性中心城市功能，推进市域一体发展，打造长江中游区域性综合交通枢纽、现代物流中心和公共服务中心。推进大冶湖生态新区建设大开发、大建设、大提升，全面建成核心区，主体功能设施基本完善，打造支撑黄石未来发展的产业新区、城市新区、生态新区。到“十三五”末，全市建成区面积超过150平方公里，城镇化率超过65%，城市辐射带动和高端要素集聚功能显著提升，城市空间布局进一步优化。推动物质文明和精神文明协调发展，努力建设文化强市，成功创建国家历史文化名城。

(三)坚持绿色发展，展现美丽黄石新形象。以生态立市为引领，把绿色发展理念融入经济社会发展全过程，促进人与自然和谐共生。以绿色经济为支撑，大力推进节能减排，合理开发利用资源，加快构建节约化、循环化、低碳化绿色产业体系。以绿色惠民为根本，大力实施治山、治水、治气、治土工程，深入开展“绿满黄石”行动，让黄石的天更蓝、地更绿、水更净、空气更清新。以生态自觉为保障，全面落实绿色发展主体责任，建立每年由一个县(市)区举办一届市级园博会机制，推动绿色风尚养成。到2020年，森林覆盖率达到36%，城区空气质量优良率达85%，主要湖泊水体水质基本达到环境功能区要求，成功创建全国文明城市、国家卫生城市、国家环保模范城市、国家森林城市和国家生态市。

(四)坚持开放发展，增创开放型经济新优势。积极抢抓国家“一带一路”、长江经济带和长江中游城市群建设机遇，依托长江黄金水道，以大路网、大港口、大物流、大通关建设为重点，建设长江经济带重要战略节点城市。坚持引资和引技引智并重、引进来和走出去并联，强力推进产业链招商，引导黄石制造、黄石服务“走出去”，不断提高开放型经济发展水平。创新开放合作的新模式、新路径、新机制，加强区域战略合作，优化经济发展环境，提高投资贸易便利化水平，努力打造内陆沿江开放高地和湖北东向开放桥头堡。力争“十三五”期间，实际利用外资和外贸出口年均分别增长10%以上。

(五)坚持共享发展，创造美好幸福新生活。坚持普惠性、保基本、均等化、可持续方向，突出抓好义务教育、就业服务、社会保障、基本医疗和公共卫生、文化惠民等工作，增加公共服务供给，努力让人民群众有更多获得感。围绕“精准扶贫、不落一人”目标，推进精准扶贫、精准脱贫，坚决打赢脱贫攻坚战。坚持居民收入增长和经济增长同步、劳动报酬提高和劳动生产率提高同步，持续增加城乡居民收入。推进平安黄石建设，加强和创新社会治理，加快完善党委领导、政府负责、社会协同、公众参与、法治保障的社会治理格局。积极应对人口老龄化，保障妇女和未成年人权益，做好特殊对象和人群的人文关怀，让全市人民共享改革发展新成果，早日过上更加幸福美好的小康生活。

三、2016年工作安排

2016年，是“十三五”规划的开局之年，是全面建成小康社会决胜阶段的首战之年，也是我市抢抓机遇、实现弯道超越的关键之年，做好今年工作，责任重大、意义深远。面对复杂严峻的发展形势和经济下行持续加大的压力，我们将顺应大势，抢抓机遇，提振精神，扎实工作，坚持逆势而进、负重发展，从需求侧和供给侧两端发力，确保开好局、起好步。

2016年经济社会发展的主要预期目标是：地区生产总值增长8%左右；规模以上工业增加值增长7%；固定资产投资增长16%左右；社会消费品零售总额增长12%左右；外贸出口总额增长11%；地方公共财政预算收入增长8%；城镇常住居民人均可支配收入增长9%；农村常住居民人均可支配收入增长10%；居民消费价格指数控制在3.5%以内；城镇登记失业率控制在4.5%以内；人口自然增长率控制在10‰以内。全面完成省下达的节能减排任务。

围绕上述目标，重点抓好六个方面的工作：

(一)以振兴黄石制造为引擎，加快推进产业转型升级

大力发展先进制造业。深入实施振兴“黄石制造”行动，加快工业绿色转型发展。坚持集中、集群、集约发展，支持欣兴电子、定颖电子等项目建设，超常规发展电子信息、节能环保、新材料等新兴产业。坚持培优、培特、培精发展，支持三丰机器人、光洋应用、博凯医药等项目建设，培育壮大装备制造、智能模具、汽车零部件、生物医药等优势产业。坚持改造、延伸、集群发展，支持新冶钢模铸精品生产线改造、大冶有色机械装备制造基地等项目建设，做优做强传统产业。着力盘活武汉重冶、晟祥铜业等企业，推进相关企业兼并重组，消化过剩产能。全面落实支持企业发展各项优惠政策，切实降低企业运行成本，大力扶持实体经济过冬。持续开展“千名干部进千企”活动。支持举办优势产业产品展览会。深入实施中小企业成长工程，力争新进规企业100家以上。

突破性发展现代服务业。出台促进现代服务业发展政策。加快现代金融业发展，实施市域金融工程，积极引进各类金融机构，大力发展普惠金融、乡镇金融，构建多样化金融服务体系。积极对接省长江产业投资基金，建立“1+N”产业投资引导基金，引导金融机构加大对重点产业的支持力度，推动金融与产业深度融合。深化政银企合作，引导金融机构对实体经济不抽贷、不压贷、不断贷，力争全年新增小微企业贷款30亿元以上。积极争取棋盘洲保税物流园外汇便利政策。大力发展商贸物流业，加快实施工业企业主辅分离，促进研发、设计、现代物流等产业发展。支持华中矿产品交易中心、亿赞普等平台做大做强。加快团城山商圈、万达广场商圈建设。健全农产品流通规划体系，完善农村商贸流通网络，加快推进电商进农村。以“智慧城市”建设为契机大力发展信息服务业，建成全市统一的大数据平台，力争在数据集成、行政审批、便民一卡通的应用上实现突破。按照无线城市的要求，实现中心城区免费wifi全覆盖。努力开通国际宽带出口。大力发展文化旅游业，以工业遗址开发建设为依托，带动乡村生态、宗教文化、红色文化旅游发展，建成东方文化园，推动商旅文深度融合发展。大力发展健身休闲、竞赛表演、场馆服务、中介培训等体育服务业和健康养老业。

加快发展现代农业。转变农业发展方式，加快推进黄石国家农业科技园区建设，建成一批绿色农产品生产基地。大力培育新型农业经营主体，加快发展农产品加工业，支持家庭农场、农民合作社、种养大户、农业产业化龙头企业发展，新增省级农业产业化龙头企业2家、规模以上农产品加工企业10家、新型经营主体100家以上。大力推进规模化、标准化、绿色化生产，新增无公害、绿色、有机品牌5个以上。全面提升农业机械化水平。大力发展观光农业、休闲农业，繁荣农村经济。

强力推进产业链招商和项目建设。完善招商引资政策，围绕我市产业规划和发展布局，紧盯世界500强、国内100强等行业龙头企业，开展产业链招商、驻点精准招商和专业机构招商，着力引进一批重大项目，力争全市新注册重点项目150个以上，实际利用外资3亿美元以上。实行重点项目专题推进机制，坚持一月一调度、一月一评比、一月一通报，抓好269个亿元以上重大项目实施，加大对上争取力度，确保固定资产投资完成1600亿元。加强园区平台建设，推进黄石开发区超常规发展。抓好黄石大冶湖高新园区创建国家级高新区工作。统筹推进省级园区发展，加快配套设施建设，增强园区服务功能和项目承载能力。

加快创新驱动发展。积极申报国家创新型试点城市。健全产学研合作长效机制，鼓励企业与高校院所共建研发机构，支持新冶钢、劲牌等企业申报国家级工程技术研究中心。继续抓好科技企业培育工作。完善科技成果转化服务和激励机制，建立科技成果信息发布和共享平台，推动50项以上科技成果在我市转化。加大创新链建设力度，推动创新链与产业链融合。深入实施知识产权战略，强化企业主体地位，促进知识产权创造运用，力争发明专利申请量1100件以上，专利授权量1000件以上。开展全国质量强市示范市创建，加快建设区域品牌，形成一批知名品牌和优势企业。

(二)以大冶湖生态新区为龙头，加快建设特大城市

全力推进大冶湖生态新区建设。加快大冶湖两座跨湖大桥、“两纵一横”道路、核心区主干道地下综合管廊建设，确保两座跨线立交桥建成通车。加快园博园、矿博园、奥林匹克体育公园，生态馆、地质馆、城市综合馆，市民中心、鄂东医疗中心、社会福利中心“三园、三馆、三中心”建设，着力完善新区功能。按照产城融合思路，建设一批公共服务设施，努力打造设施齐全、宜居宜业生态新区。

着力提升城市功能。以建设“海绵城市”、“韧性城市”为契机，坚持规划、建设、管理三结合，加强城市设计，提倡城市修补，着力在提高城市发展持续性、宜居性上下功夫。维修改造城市道路2万平方米。推进鼎丰闸泵站、磁湖区域污水管网、新港污水处理厂建设，完成中心城区排渍泵站、河西地区排洪港、河西污水处理厂、下陆铁山污水处理配套管网建设。加快大冶有色改水、黄金山供水、河西供水工程建设，完成城网供水二期、王英水库备用水源引水工程建设。新建天然气管网30公里。开工建设城市轨道交通项目。完成黄石电动汽车充电设施布局专项规划。在中心城区规划建设一批立体停车场。

大力推进新型城镇化建设。积极对接国家和省“十三五”规划，推进城乡规划全覆盖和“多规合一”，加快编制城市绿地系统和绿线专项规划、特色村落与传统村落风貌保护规划。抓好黄石城区与大冶市区设施对接、一体建设、同城发展。支持阳新城东新区建设，打造市域副中心。推进城镇群发展。认真组织省市“四化同步”示范镇试点工作，推进10个中心镇和100个中心村建设，完成金牛、还地桥、富池等镇污水处理厂和配套收集管网建设，确保投入使用。

(三)以黄石新港多式联运为切入点，积极融入长江经济带

加快港口建设。按照“亿吨大港”的要求，以黄石新港为龙头，全方位加强与盐田港集团合作，加速推进黄石新港一、二期工程建设，统筹实施阳新兴国、富池作业区综合码头工程。加快黄石花湖物流中心、黄石(大冶)罗桥物流园等项目建设，完善运输、仓储、配送等物流链条，加快形成以港口为依托的多式联运物流网络。

加快口岸通关建设。完善棋盘洲保税物流中心配套功能，着力推进综保区筹备工作，加强电子口岸建设，积极融入长江流域通关一体化，不断提高通关效率。加快“国家级出口基地”建设，大力培育电子信息、装备制造出口产业集群，不断提高外贸出口规模和水平。

加快交通枢纽建设。加快推进黄阳快速路、港湖至保安公路、刘仁八至金牛公路建设，力争汪仁至阳新县城段建成通车。抓好黄富沿江公路等项目前期工作。建成“两区两带”精准扶贫公路、兴国至富池和龙港至洋港公路。改造县乡公路50公里、通村公路300公里、农村公路桥梁20座。建成武九客专黄石段。抓好棋盘洲长江公路大桥及连接线、武穴长江公路大桥、鄂州至咸宁高速黄石段、新港货运支线、团城山客运枢纽站等项目建设，启动武九客专大冶北站、阳新南站公铁联运客运枢纽站前期工作，进一步巩固黄石交通枢纽地位。

加快临江产业布局。建立临江产业发展协作机制，完善企业“退城入园、临江发展”政策，鼓励城区优势企业、重点项目优先在临江布局，抓好宝钢、新兴际华搬迁项目建设，着力打造临江经济走廊。深度对接武汉，抓好跨江、跨区域联动发展，主动承接长三角、中三角产业转移，不断提升参与长江流域合作开放水平。

(四)以全面深化改革为动力，不断激发发展潜能

深化要素改革。按供给侧结构改革要求，优化资源配置，努力提高全要素生产率。加强资本市场建设，建立“改制、入库、托管、挂牌、上市”新机制，推动企业上市再融资。深化市级投融资平台改革。健全政府和社会资本合作机制。提高保险业对地方经济发展的贡献率。加快信用征信体系建设，努力构建科学准确、覆盖面广的信用征信体系，促进我市金融生态环境进一步优化，预防和化解金融风险。深化土地和矿产资源利用改革，完善矿业投融资平台和矿产资源管理机制，调整矿业开发布局和结构，提高矿产资源开发利用效益。深入开展工矿废弃地复垦利用试点和增减挂钩试点。创新人才及人力资源利用机制，加大人才引进力度，加强人才与主导产业对接，继续抓好“东楚英才计划”、“新兴支柱产业人才扶持计划”，培养更多的工匠和新兴技能人才。

深化行政审批制度改革。加强权力清单、负面清单、责任清单的实施和监管。推进“容缺受理、并联审批、优化流程、全程代办”改革，在黄石开发区开展行政审批改革试点，实行“一枚公章管审批”。建设全市政务服务“一张网”，打造办事快捷、管理高效、服务便利的智慧政府平台，不断强化制度供给。

创新发展体制机制。抓好全国内贸流通体制改革发展综合试点，探索建立“城与乡”、“内外贸”、“商旅文”融合发展新机制，着力打造法治化营商环境和统一高效的流通管理体制。深入推进大众创业、万众创新，打造一批市场化、专业化、网络化、多元化众创空间和大学生创新创业示范园。创新财政管理机制，加大政府性基金预算、国有资本经营预算与一般公共预算的统筹力度，进一步理顺市与城区的事权和支出责任。深化农村综合配套改革，积极开展农村集体经济组织产权制度改革试点。大力推进农垦改革和供销合作社综合改革。加快推进不动产统一登记和征地制度改革，探索建立城乡统一的建设用地市场。继续深化国企改革。

(五)以承办省园博会、矿博会为抓手，着力推进绿色发展

抓好生态环境治理。推进山体治理，全面完成“五边三化”三年行动目标任务，完成矿山地质环境治理重点工程三期任务。坚持打好大气污染防治攻坚战，进一步改善空气质量。落实国务院《水污染防治行动计划》，加强以长江黄石段、富河、王英水库为重点的饮用水源地环境监管，推进以磁湖为重点的亚行水污染综合治理、“三湖一河一库”治理等重大项目，强力推进大冶湖拆围工作，完成铁金港流域污水治理任务，切实改善水环境质量。实施重金属污染防治重点工程。支持阳新申报国家重点生态功能区。

强化节能减排。全面加强重点领域节能管理，抓好重点能耗企业能源对标工作，推动重大节能技术产品规模化生产和应用，继续淘汰一批黄标车，全面落实居住建筑节能设计标准。深入推进工程减排、结构减排和管理减排，加大城区燃煤锅炉淘汰力度，加快实施畜禽养殖污染防治等一批重点项目。

统筹推进“五城同创”。深入实施全国文明城市创建三年行动计划，加强志愿者队伍建设，广泛开展群众性精神文明创建活动。深入推进国家卫生城市创建工作，确保2016年通过省级初评，力争通过全国爱卫会考核评估。统筹推进“绿满黄石”行动与森林城市创建，完成植树造林16万亩。启动创建国家森林城市工作。高标准编制国家生态市创建规划，全力推进省级生态县创建工作，建成一批省、市级生态村。认真做好国家环保模范城市创建工作。积极创建国家生态文明先行示范区。大力实施绿化、净化、美化、畅化“四化”工程，完善城市综合管理、部门联合执法等机制，提升城市建设管理水平。

加快建设美丽乡村。加强农田水利薄弱环节建设，抓好农村饮水安全、病险水闸整险、大型泵站更新改造、农村水电改造等重点项目。深入推进新农村建设，完善乡镇环卫配套设施和农村垃圾一体化收集处理体系，健全村庄保洁长效机制，确保90%以上生活垃圾无害化处理。

做好省园博会、矿博会筹办工作。统筹推进园区配套工程、城市园林绿化等4大类80个重点项目建设，力争出精品、出亮点。认真组织好八大重点活动，努力把本届园博会办成有特色、有影响，展示“转型黄石?灵秀湖北”的精彩盛会。以园博会为契机，精心组织一系列城市形象宣传推介活动，着力营造“精彩园博、人人参与”的浓厚氛围。

(六)以保障和改善民生为目的，切实增进人民福祉

实施精准扶贫。坚持“四看”标准，加快推进农村土坯房、厨房和厕所改造，拓宽贫困农民增收渠道，全面提高贫困地区就学、就医和社会保障水平。大力推进贫困村通湾通组公路、农村电网、宽带进村入户等建设，改善贫困地区基础设施条件。加快推进老区库区精准扶贫示范带、美丽乡村展示带建设，着力打造“两镇一区”精准扶贫样板带、美丽乡村体验带。抓好异地扶贫搬迁工作。支持大冶率先实现全面脱贫。

大力推进棚改民生工程。按照“三年计划”部署，抢抓国家棚户区改造的政策机遇期，建立多平台多银行全方位融资体系，破解资金瓶颈。加大货币化安置力度，建立代筹、代购、代建协调配套的安置格局，扎实推进城镇棚户区、危旧房和城中村改造，确保开工各类保障性安居工程20000套(户)，基本建成各类保障房7591套(户)，实现分配入住16726套(户)，货币化安置率达到80%以上。通过棚改，加大房地产市场去库存力度。

完善社会保障。突出抓好新生代青年群体就业工作，畅通就业信息渠道，根据市场需求加大就业培训力度，缓解劳动力供需矛盾和就业结构性矛盾，城镇新增就业5万人以上。实施机关事业单位养老保险制度改革。加快落实以个人身份参加企业职工基本养老保险政策，引导被征地农民分类有序参加基本养老保险。扎实推进全民医保体系建设，做好进城落户农民参加基本医疗保险和关系转移接续工作，新农合参合率稳定在99%以上，新农合政策范围内住院费用支付比例稳步提高。巩固完善大病保险制度，进一步扩大省内异地就医即时结算范围。加强各项社会救助制度衔接，实现农村低保线和扶贫线“两线合一”，突出抓好农村五类重点对象帮扶，做到兜底线、救急难。加快养老服务信息化建设，支持民营养老服务业发展，组建鄂东医养集团。促进残疾人就业。保障妇女儿童权益。支持发展慈善事业。推进公共法律服务体系建设，加大法律援助力度，维护社会公平正义。

协调发展社会事业。抓好农村幼儿园设施建设，加快普及学前教育。创建30所标准化学校，推动义务教育优质均衡发展。完善普通高中经费保障机制，促进高中教育内涵发展。安排财政专项资金，对留住毕业生在黄就业的市属职业院校、高校给予奖励，推动职业教育与我市产业发展相适应。支持黄石八中建设全国领航学校。支持湖师文理学院新校区建设。认真实施华新水泥厂旧址文物保护和汉冶萍煤铁厂矿修缮工程，着力推进国家历史文化名城申报和黄石矿冶工业遗产申报世界文化遗产工作。实施文化惠民工程，完成市图书馆旧馆改扩建，确保市群艺馆新馆投入使用。建设农村文化礼堂500家，实现全覆盖。繁荣发展文艺创作、新闻出版、广播影视等事业。抓好农村古民居保护。深化医疗卫生体制改革，优化医疗资源配置，尽量让群众少花钱、更方便、看好病。大力支持鄂东医疗集团改革发展。加快人口健康信息平台建设。落实“健康黄石”全民行动，实现健康管理联合体全覆盖。依法实施全面两孩政策，加强出生人口性别比控制，促进人口均衡发展。全力筹备及备战省第十五届运动会，抓好磁湖水上运动中心、国家登山健身步道等项目建设，举办第三届磁湖户外运动节暨全国半程马拉松邀请赛。

创新社会治理。深入推进全国社区治理和服务创新实验区建设。加强城乡网格化服务管理，实现全市城乡网格化建设全覆盖。推进信访制度改革，强化人民调解、行政调解、司法调解衔接配合，完善第三方化解机制。加快市群众信访服务中心建设。健全立体化社会治安防治体系，基本建成社区防控、街面巡控、虚拟社会网络防控等九张网。加快平安黄石建设，扩大平安社区(村)覆盖面，提升群众安全感满意度。贯彻新《安全生产法》，持续抓好打非治违及重点行业、重点领域专项整治，确保全市安全生产形势稳定。构建属地管理、分级负责、多级联动、严密高效的食品药品监管体系，扎实推进“餐桌污染”治理，建设“食品放心工程”，让人民群众吃饭安心、用药放心。完善各类应急救援体系，提高公共安全管理和防灾救灾减灾能力。启动“七五”普法工作。抓好民族宗教工作。大力支持国防后备力量建设，推进军民融合发展，争创省“双拥”模范城。

与此同时，加快建设“开明、精明、廉明、亲民”政府，自觉接受人大及其常委会的法律监督、工作监督，自觉接受人民政协的民主监督，主动接受社会监督、舆论监督，积极听取民主党派、人民团体和各界人士的意见建议，认真办理人大代表建议和政协提案，加强审计监督，完善政府法律顾问制度，依法全面履行政府职能，加快推进法治政府建设。严格落实政府系统党风廉政建设责任制，认真落实“一岗双责”，严肃查处违法违纪案件。加强政府行政效能建设，着力解决慢作为、不作为问题，严格执行行政问责制度，确保政令畅通、令行禁止，切实提高政府执行力和公信力。

各位代表，蓝图已经绘就，奋斗谱写辉煌。让我们紧密团结在以习近平同志为总书记的党中央周围，在中共黄石市委的坚强领导下，以令行禁止的大局观念、雷厉风行的效率意识、迎难而上的担当精神、一抓到底的工作狠劲，坚定信心、保持耐心、树立雄心，奋力实现“十三五”良好开局，为率先全面建成小康社会而努力奋斗!
